# Antimicrobial activity of biogenically produced spherical Se‐nanomaterials embedded in organic material against *Pseudomonas aeruginosa* and *Staphylococcus aureus* strains on hydroxyapatite‐coated surfaces

**DOI:** 10.1111/1751-7915.12700

**Published:** 2017-02-23

**Authors:** Elena Piacenza, Alessandro Presentato, Emanuele Zonaro, Joseph A. Lemire, Marc Demeter, Giovanni Vallini, Raymond J. Turner, Silvia Lampis

**Affiliations:** ^1^ Biofilm Research GroupDepartment of Biological SciencesUniversity of Calgary2500 University Dr NWCalgaryABT2N 1N4Canada; ^2^ Environmental Microbiology LaboratoryDepartment of BiotechnologyUniversity of VeronaStrada Le Grazie 1537134VeronaItaly

## Abstract

In an effort to prevent the formation of pathogenic biofilms on hydroxyapatite (HA)‐based clinical devices and surfaces, we present a study evaluating the antimicrobial efficacy of Spherical biogenic Se‐Nanostructures Embedded in Organic material (Bio Se‐NEMO‐S) produced by *Bacillus mycoides* SelTE01 in comparison with two different chemical selenium nanoparticle (SeNP) classes. These nanomaterials have been studied as potential antimicrobials for eradication of established HA‐grown biofilms, for preventing biofilm formation on HA‐coated surfaces and for inhibition of planktonic cell growth of *Pseudomonas aeruginosa* NCTC 12934 and *Staphylococcus aureus* ATCC 25923. Bio Se‐NEMO resulted more efficacious than those chemically produced in all tested scenarios. Bio Se‐NEMO produced by *B. mycoides* SelTE01 after 6 or 24 h of Na_2_SeO_3_ exposure show the same effective antibiofilm activity towards both *P. aeruginosa* and *S. aureus* strains at 0.078 mg ml^−1^ (Bio Se‐NEMO_6_) and 0.3125 mg ml^−1^ (Bio Se‐NEMO_24_). Meanwhile, chemically synthesized SeNPs at the highest tested concentration (2.5 mg ml^−1^) have moderate antimicrobial activity. The confocal laser scanning micrographs demonstrate that the majority of the *P. aeruginosa* and *S. aureus* cells exposed to biogenic SeNPs within the biofilm are killed or eradicated. Bio Se‐NEMO therefore displayed good antimicrobial activity towards HA‐grown biofilms and planktonic cells, becoming possible candidates as new antimicrobials.

## Introduction

In the last 20 years, the potential to use nanoparticles (NPs) as antimicrobial agents has been evaluated (Ankamwar *et al*., 2005). Primarily, the focus was to synthesize NPs using various chemical methods. However, both the high cost of production and presence of toxic by‐products generated a demand for novel methods to synthesize NPs (Ankamwar *et al*., 2005). Biological systems such as plants, fungi and bacteria have the capacity to convert several toxic metal ions into less toxic forms including metal precipitants or NPs (Suresh *et al*., 2004; Bhainsa and D'Souza, 2006; Song *et al.,* 2009). Thanks to the potential technological importance of such NPs, research interest has been focused on the use of these organisms to produce NPs with eco‐friendly and ‘green synthesis’ methods (Ingale and Chaudhari, 2013). One of the first classes of biogenic NPs to be evaluated was silver NPs (AgNPs), due to the demonstrated antimicrobial ability of metallic silver (Ag; Dos Santos *et al*., 2014). Generally, biogenic AgNPs are produced using fungal cultures able to bioaccumulate metals and synthesize NPs, which are excreted outside cells using their filaments (Srivastava and Mukhopadhyay, 2015). Some commonly used fungi for AgNP production are *Verticillium* (Mukherjee *et al*., 2001), *Aspergillus flavus* (Vigneshwaran *et al*., 2007) and *Fusarium oxysporum* (Ahmad *et al*., 2003). AgNP synthesis using bacterial cell extracts from *Lactobacillus acidophilus* (Rajesh *et al*., 2015) *or Corynebacterium glutamicum* (Gowramma *et al*., 2015) has also been investigated.

Recently, selenium NPs (SeNPs) has emerged as a new class of potential antimicrobial agents. Selenium is an essential micronutrient in biologic systems; it has anticancer and antimicrobial properties, antioxidant effects and modulation functions for the immune system (Sadeghian *et al*., 2012). Despite these roles, selenium can be found in the environment as toxic forms, such as selenate (SeO_4_^2−^), selenite (SeO_3_^2−^) and selenide (Se^2−^) (Lampis *et al*., 2014). Toxicity of these selenium anions depends on their mobilization and availability in soils and water, improving the possibility of exposure for humans and animals (Lampis *et al*., 2014). In this respect, efforts have been made to identify a non‐toxic form of selenium useable in biomedical applications (Wang *et al*., 2013). Recently, it has been established that SeNPs have higher biocompatibility and lower toxicity in humans compared with bulk selenium (Lampis *et al*., 2014). Additionally, SeNPs have unique physical and chemical properties due to their large surface–volume ratio, large surface energy, spatial confinement and reduced imperfections (Stroyuk *et al*., 2008). In this regard, SeNPs possess adsorptive ability, oxidation functions and marked biological reactivity, including antihydroxyl radical efficacy (Lampis *et al*., 2014). SeNPs are normally synthesized by chemical reduction of selenite or selenous acid by reducing agents such as glutathione (GSH), hydrazine, sodium borohydride (NaBH_4_), stannous chloride SnCl_2_, L‐cysteine, ascorbic acid, sodium thiosulfate (Na_2_S_2_O_3_) and SDS (Stroyuk *et al*., 2008). These techniques are unfavourable as they produce particles that are subject to photocorrosion (Dobias *et al*., 2011). Yet, it is now possible to produce SeNPs of various compositions, sizes and morphologies using bacteria (Lampis *et al*., 2014). A number of bacterial species, residing in diverse terrestrial and aquatic environments, are resistant to selenium oxyanions and possess the ability to reduce selenite and selenate into its less bioavailable elemental form (Se^0^; Stolz *et al*., 2006). This process occurs through both enzymatic and non‐enzymatic mechanisms, leading to the formation of SeNPs that are deposited inside the cell periplasm or excreted (Kessi *et al*., 1999; Oremland *et al*., 2004; Dhanjal and Cameotra, 2010).

One of the most clinically sought properties of SeNPs is their ability to inhibit pathogenic biofilm formation (Wang and Webster, 2012). Most bacteria have the innate ability to populate, as a biofilm, a vast array of surfaces, including those where sterility is of paramount importance to human health, as food‐processing facilities, dental hygiene equipment and medical devices to name a few (Harrison *et al*., 2004). Two of the most diffuse and harmful bacteria able to grow as biofilms responsible for human pathogenic infections are *Staphylococcus aureus*, which is a Gram‐positive bacterium that causes many serious infections in surgical wounds, bloodstream or in the lungs (Alhede *et al*.,^2009^), and *Pseudomonas aeruginosa*, which is a Gram‐negative bacteria recognized as one of the most important nosocomial opportunistic pathogens, affecting the urinary tract, respiratory system, soft tissue, bone and joint tissue (Alhede *et al*., 2009). Of particular interest here are biofilms of these species, which are able to grow on artificial orthopaedic implants made of hydroxyapatite (HA) – causing severe infections (Kolmas *et al.,* 2015a). HA is a naturally occurring mineral formed by calcium and phosphate ions – Ca_5_(PO_4_)_3_(OH) – with a recognizable crystalline structure (Elliot, 1994). HA is also the principal component of bones and teeth in humans and animals and is therefore used regularly in orthopaedic surgery and for the replacement of teeth (Gong *et al*., 2015). To prevent bacterial contamination and subsequent infection, research efforts have focused on developing medical devices and implants out of materials modified with antimicrobial agents such as antibiotics or metallic compounds that are able to kill bacterial cells, or inhibit their growth, without being in general toxic to surrounding tissues (Kolmas *et al.,* 2015a; Lim *et al*., 2013; Harrison *et al*., 2004). However, with the broad use and abuse of antibiotics, the emergence of bacterial resistance to these antimicrobials has become one of the greatest health challenges worldwide. Moreover, the use of metallic compounds as coating can cause severe problems, such as ion release in human body and development of several side‐effects (Crobb and Schmalzereid, 2006). Indeed, some metal ions can undergo corrosion processes, activating immunological responses and cytotoxic and genotoxic effects (Jacobs *et al*., 1994; Merritt and Rodrigo, 1996). Furthermore, the ionic species present in HA are normally subject to substitution processes that can dramatically change the structure and properties of HA itself (Kolmas *et al*., 2015b).

Recently, to overcome both bacterial resistance to antibiotics and the side‐effects of metallic compounds, new antimicrobial technologies for HA‐based implants have been investigated, such as the use of nano‐sized HA conjugated with chemical NPs known for their antimicrobial ability (Kolmas *et al.,* 2015b). In this regard, due to the peculiar chemistry of nanomaterials as compared to one of the bulk ions, the use of NPs as antimicrobial agents could constitute an alternative choice to the use of metallic ions. Specifically, chemically synthesized SeNPs were already evaluated as possible doping for HA‐based implants, thanks to either their antimicrobial or anticancer properties (Kolmas *et al*., 2015b; Wang *et al*., 2016). Selenium also plays a crucial role in bone growth and proliferation of osteoblasts (Kolmas *et al*., 2015b), and SeNPs could prove to be an added value as an antimicrobial coating for HA‐based implants.

In this study, we evaluated the antimicrobial and antibiofilm efficacy of biogenic SeNPs as a useful and alternative approach to chemically synthesized SeNPs, which are already described for their ability to inhibit biofilm proliferation (Wang and Webster, 2012). The antimicrobial properties of both chemical and our biogenic Se‐nanomaterials were studied towards *P. aeruginosa* and *S. aureus* biofilms grown onto HA‐coated surfaces. Biogenic SeNPs were produced by exposing *Bacillus mycoides* SelTE01 for either 6 or 24 h to Na_2_SeO_3_. This strain is a Gram‐positive bacterium isolated from the selenium hyperaccumulator plant *Astragalus bisulcatus* described for its ability to reduce selenite oxyanion into its elemental form as SeNPs (Lampis *et al*., 2014). Chemical SeNPs were synthesized using L‐cysteine (Li *et al*., 2010) or ascorbic acid (Zhang *et al*., 2004) as reducing agents.

## Results

### Characterization of SeNPs

**Figure 1:**
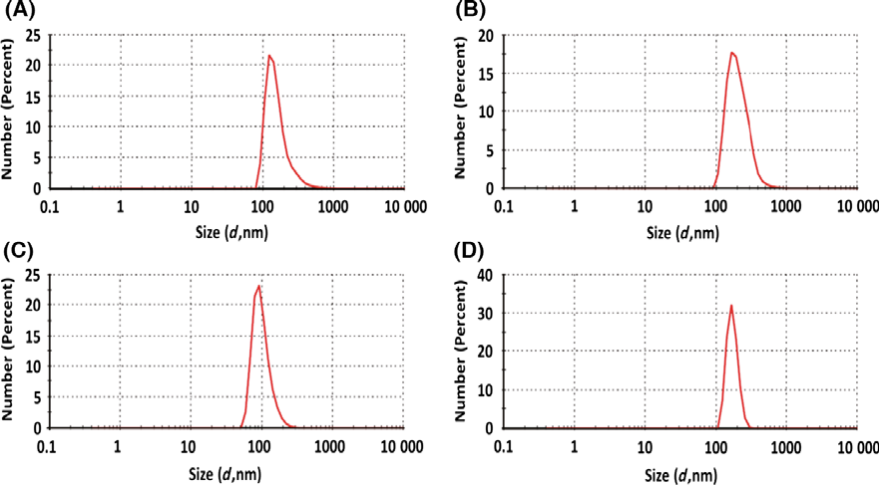
Dynamic light scattering (DLS) analysis of biogenic SeNPs produced by *Bacillus mycoides* SelTE01 after 6 (A) or 24 h (B) of Na_2_SeO_3_ exposure, and chemical SeNPs made using L‐cysteine (C) or ascorbic Acid (D).

The hydrodynamic diameter of chemically and biogenically produced SeNPs has been evaluated by dynamic light scattering (DLS) analysis (Fig. 1). Biogenic SeNPs produced by *B. mycoides* SelTE01 after 6 h of Na_2_SeO_3_ exposure are characterized by a sharp peak at 160 ± 58.6 nm (Fig. 1A), while a broad and shifted peak of 209.1 ± 79.1 nm has been detected for those obtained after 24 h of growth in the presence of the selenite precursor (Fig. 1B). DLS number size distributions of SeNPs produced using L‐cysteine (L‐cys SeNPs) or ascorbic acid (Asc SeNPs) show sharp and defined peaks at 99.8 ± 30.2 nm and 170.5 ± 64.4 nm respectively (Fig. 1C and D). Furthermore, polydispersity indexes (PDIs) of both chemically and biogenically synthesized SeNPs have been also evaluated to study the stability of NPs in solution. L‐cys SeNPs and biogenic SeNPs produced after 6 h of Na_2_SeO_3_ exposure are characterized by similar PDI values, namely 0.198 and 0.220. Moreover, comparable PDI values for Asc SeNPs (0.312) and those biogenically synthesized after 24 h of precursor exposure (0.290) have been determined.

**Figure 2:**
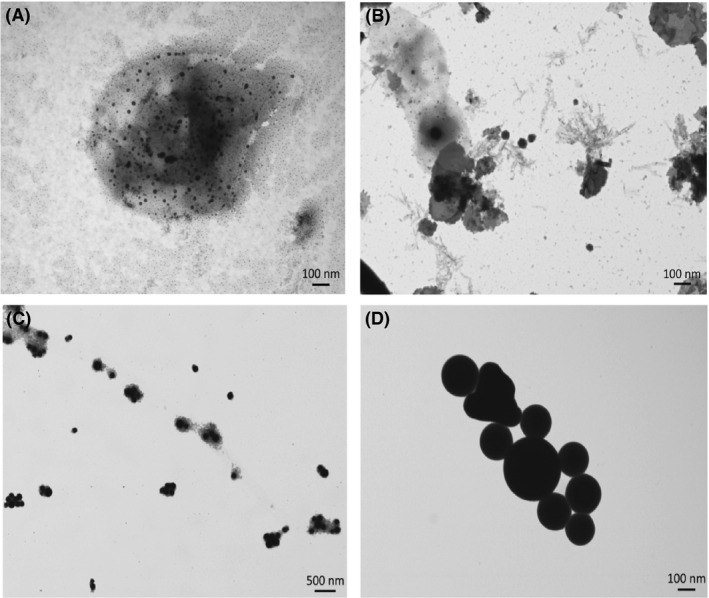
Transmission Electron Microscopy (TEM) analysis of biogenic SeNPs produced by *Bacillus mycoides* SelTE01 after 6 (A) or 24 h (B) of Na_2_SeO_3_ exposure, and chemical SeNPs made using L‐cysteine (C) or ascorbic Acid (D).

Transmission Electron Microscopy (TEM) micrographs of biogenically produced SeNPs highlighted the presence of spherical highly electron‐dense NPs different in size and embedded in a light grey and uniform matrix (Fig. 2A and B). Analysis of L‐cys SeNPs showed spherical and strongly electron‐dense NPs more uniform in size rather than those biogenically produced (Fig. 2C). Considering Asc SeNPs, TEM image showed big NPs with aggregates in the population (Fig. 2D). However, in both chemical SeNP classes, the presence of an embedding matrix has not been detected.

**Figure 3:**
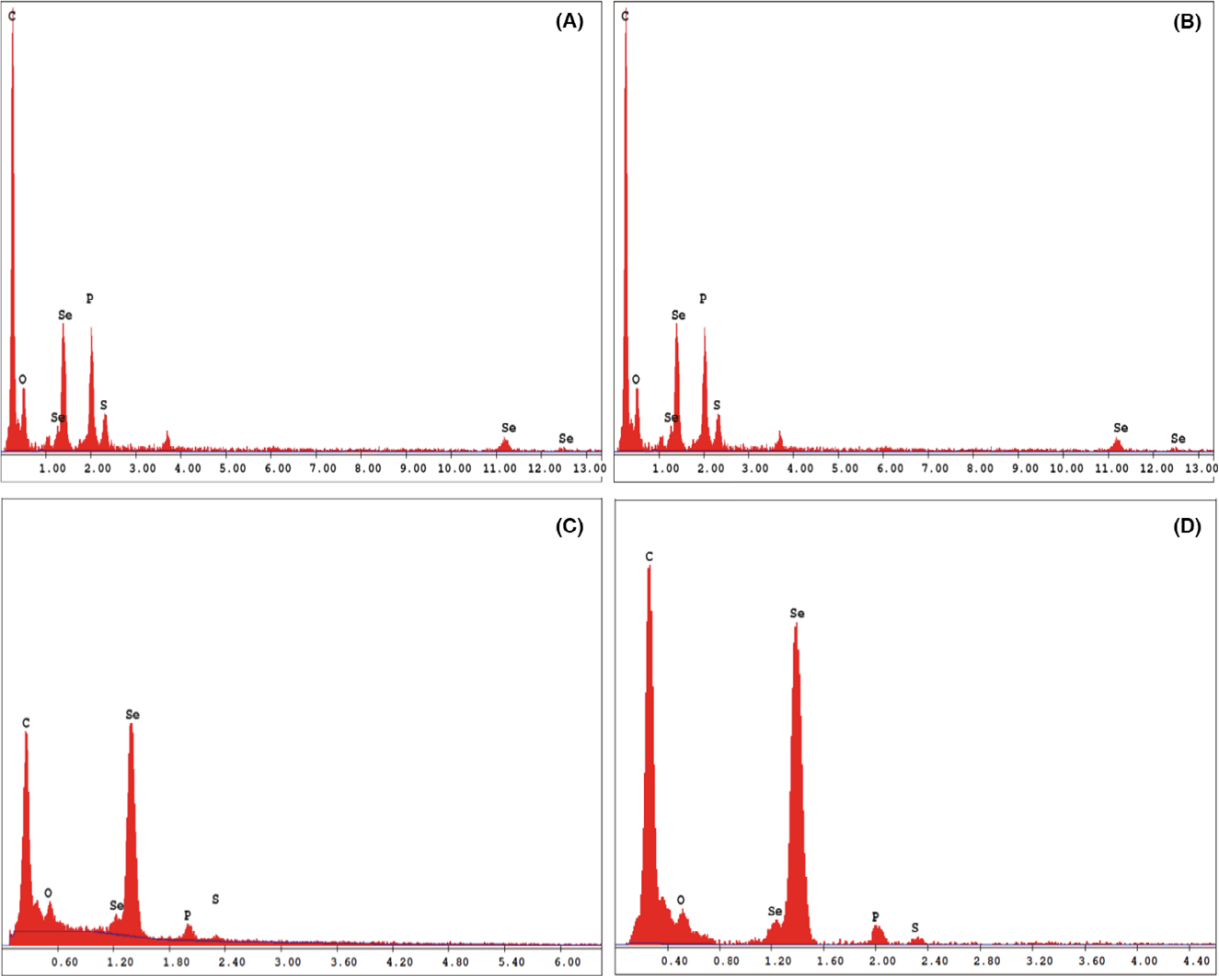
Energy‐dispersive X‐ray spectroscopy (EDX) spectra of Bio Se‐NEMO‐S produced by *Bacillus mycoides* SelTE01 after 6 (A) or 24 h (B) of Na_2_SeO_3_ exposure, and chemical SeNPs made using L‐cysteine (C) or ascorbic Acid (D).

Elemental composition of biogenically and chemically produced SeNPs has been evaluated by performing energy‐dispersive X‐ray spectroscopy (EDX). Biogenic SeNPs produced by *B. mycoides* SelTE01 after 6 or 24 h of Na_2_SeO_3_ exposure revealed the presence of the characteristic Se absorption peaks at 1.37 (SeLα), 11.22 (SeKα) and 12.49 (SeKβ) keV, while only the SeLα peak was detected in both chemically synthesized SeNPs (Fig. 3). Moreover, both biogenic and chemical SeNPs showed similar elemental composition, with the presence of selenium, carbon, oxygen, phosphorus and sulphur, but in different relative percentages (Table 1). Biogenically synthesized SeNPs showed minor differences in the detected relative percentage values of P and S, while C, O and Se were present in similar amount in both samples. Overall, the relative percentage values of carbon, oxygen, phosphorous and sulphur for biogenic SeNPs suggested the presence of organic molecules associated with the extracted SeNPs, and TEM observations confirmed the presence of a slightly electron‐dense material surrounding the particles. Thus, from here on, we refer to biogenically synthesized SeNPs as Spherical Bio Se‐Nanostructures Embedded in an Organic material (Bio Se‐NEMO‐S). Particularly, Bio Se‐NEMO‐S_6_ and Bio Se‐NEMO‐S_24_ refer to the biogenic SeNPs produced by *B. mycoides* SelTE01 after 6 and 24 h of Na_2_SeO_3_ exposure respectively.

**Table 1:** Elemental quantification (as weight relative percentage) of Bio Se‐NEMO and chemical SeNPs

	Bio Se‐NEMO‐S		Chemical SeNPs	
	Bio Se‐NEMO‐S_6_	Bio Se‐NEMO‐S_24_	L‐cys SeNPs	Asc SeNPs
Element	Weight (Rel. %)	Weight (Rel. %)	Weight (Rel. %)	Weight (Rel. %)
Selenium (Se)	9.26	9.82	31.61	16.04
Carbon (C)	75.75	80.71	60.91	76.88
Oxygen (O)	10.82	8.45	4.97	6.27
Phosphorous (P)	3.14	0.73	1.88	0.62
Sulphur (S)	1.04	0.29	0.63	0.19

Elemental quantification is expressed as weight relative percentage of the element detected in the SeNPs samples.

For both chemically synthesized SeNPs, the same elements (C, O, Se, P, S) are present yet with different relative percentage values, due to the procedure of the production, using either L‐cysteine or ascorbic acid as reducing agent (Table 1).

**Figure 4:**
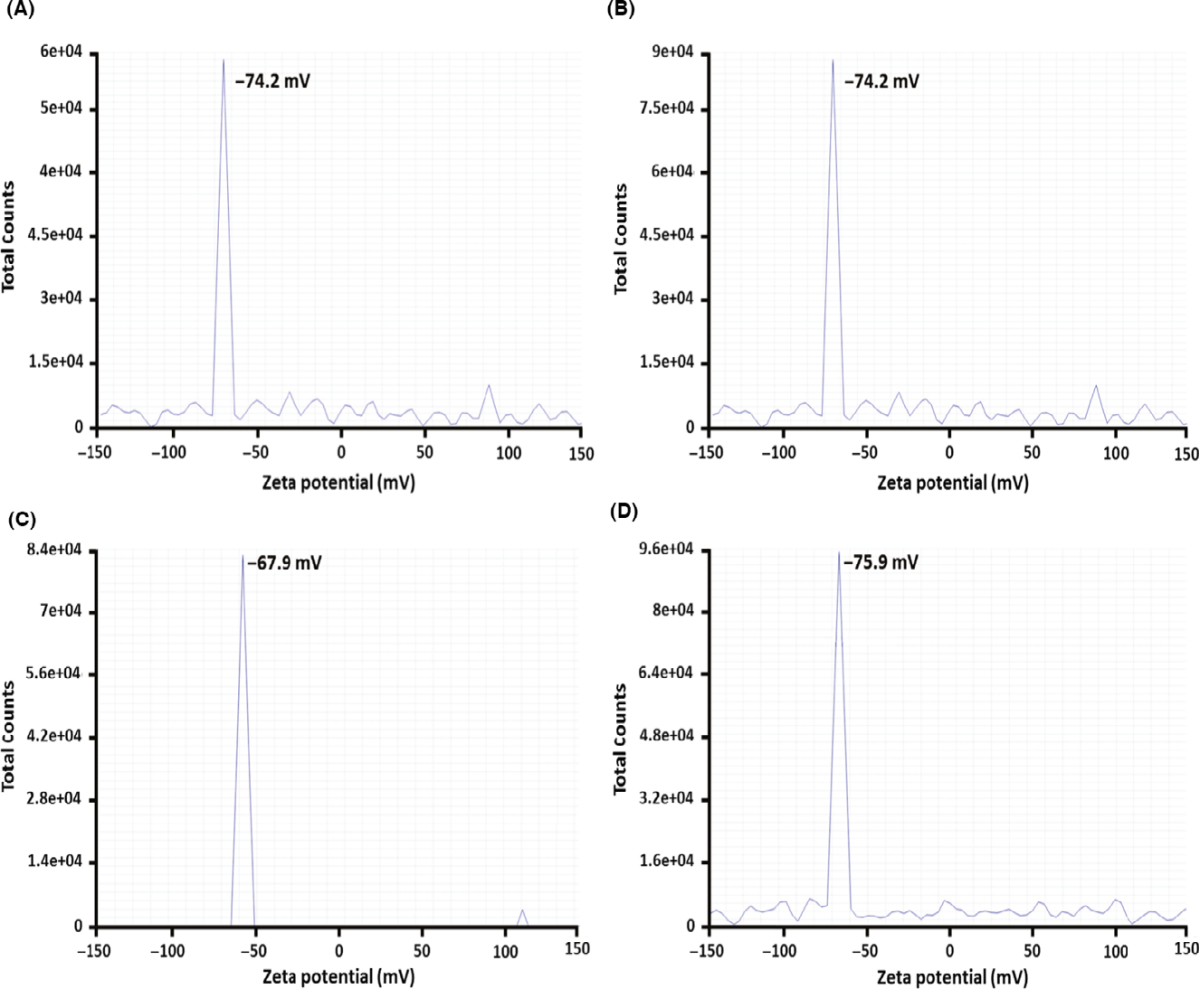
Zeta potential measurements of Bio Se‐NEMO‐S produced by *Bacillus mycoides* SelTE01 after 6 (A) or 24 h (B) of Na_2_SeO_3_ exposure, and chemical SeNPs made using L‐cysteine (C) or ascorbic acid (D).

Zeta potential measurements have been carried out to study the stability of either Bio Se‐NEMO‐S or chemical SeNPs in solution. In all four cases, a highly negative zeta potential value has been detected (Fig. 4). Particularly, both Bio Se‐NEMO‐S_6_ and Bio Se‐NEMO‐S_24_ generated the same surface charge of −74.2 mV (Fig. 4A and B), while L‐cys and Asc SeNPs showed a Z potential value of −67.9 and −75.9 mV respectively (Fig. 4C and D).

### Evaluation of the antimicrobial ability of Bio Se‐NEMO and chemical SeNPs

#### Antimicrobial activity against established biofilms

**Figure 5:**
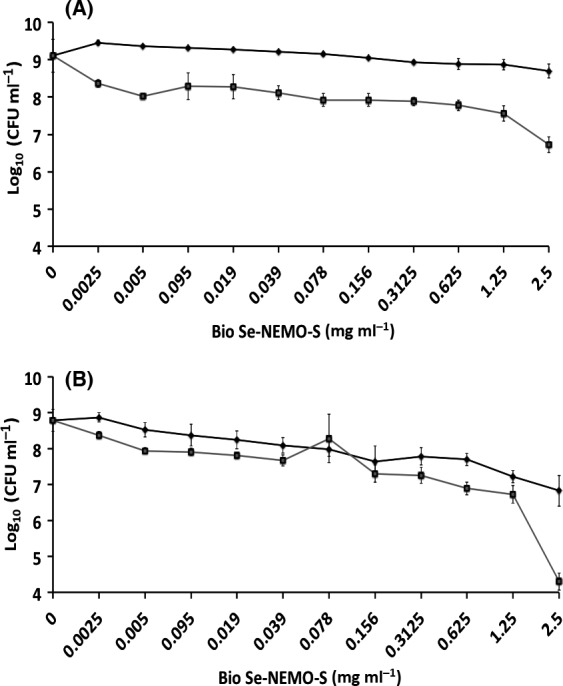
Minimal Biofilm Eradication Concentration (MBEC) assays of *Pseudomonas aeruginosa* NCTC 12934 (A) and *Staphylococcus aureus* ACTT 25923 (B) established biofilms for 24 h, and subsequently exposed for 24 h to 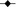 Bio Se‐NEMO‐S_6_ and 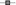 Bio Se‐NEMO‐S_24_. Error bars show the standard deviation.

**Figure 6:**
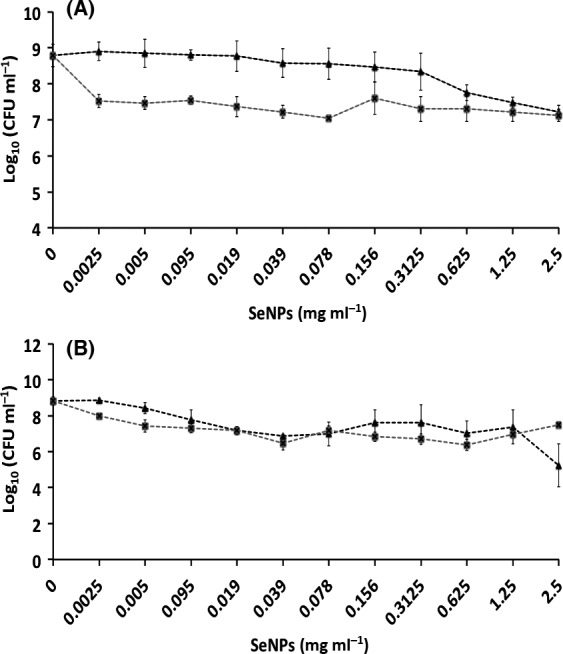
Minimal Biofilm Eradication Concentration (MBEC) assays of *Pseudomonas aeruginosa* NCTC 12934 (A) and *Staphylococcus aureus* ACTT 25923 (B) established biofilms for 24 h, and subsequently exposed for 24 h to 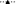 L‐cys SeNPs and 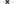 Asc SeNPs. Error bars show the standard deviation.

Eradication of an established biofilm has been evaluated growing both *P. aeruginosa* NCTC 12934 (Figs 5A and 6A) and *S. aureus* ATCC 25923 (Figs 5B and 6B) strains for 24 h prior the treatment with each tested Se‐nanomaterial using HA‐coated Calgary Biofilm Devices (CBDs) to allow the growth of biofilms (Harrison *et al*., 2010). Results are shown for both Bio Se‐NEMO‐S (Bio Se‐NEMO‐S_6_ and Bio Se‐NEMO‐S_24_) and chemical SeNPs (L‐cys and Asc SeNPs). Particularly, in the case of *P. aeruginosa* grown biofilm, Bio Se‐NEMO_24_ and Asc SeNPs showed a 2 log decrease in the cell viable count at the concentration of 2.5 mg ml^−1^, while a slight antimicrobial activity (1 log reduction) has been observed for Bio Se‐NEMO‐S_6_ and L‐cys SeNPs (Figs 5A and 6A). Considering *S. aureus* established biofilm, Bio Se‐NEMO‐S_24_ and Bio Se‐NEMO‐S_6_ affected its fitness with a 2 log reduction in the cellular population at the concentrations of 0.625 and 1.25 mg ml^−1^ respectively (Figs 5B and 6B). Moreover, Bio Se‐NEMO‐S_24_ exerted their strongest antimicrobial ability (4 log) against *S. aureus* grown biofilm at 2.5 mg ml^−1^, while the same concentration of L‐cys and Asc SeNPs determined a 2 log decrease upon the cells within the biofilm (Figs 5B and 6B). Nevertheless, in the range of tested concentrations, none of the studied Bio Se‐NEMO‐S or SeNPs classes showed a complete eradication of a pre‐formed biofilm. Thus, a Minimal Biofilm Eradication Concentration (MBEC), that is the concentration of either Bio Se‐NEMO‐S or chemical SeNPs at which there is the eradication of an already established biofilm, could not be determined.

#### Inhibition of biofilm formation and growth

**Figure 7:**
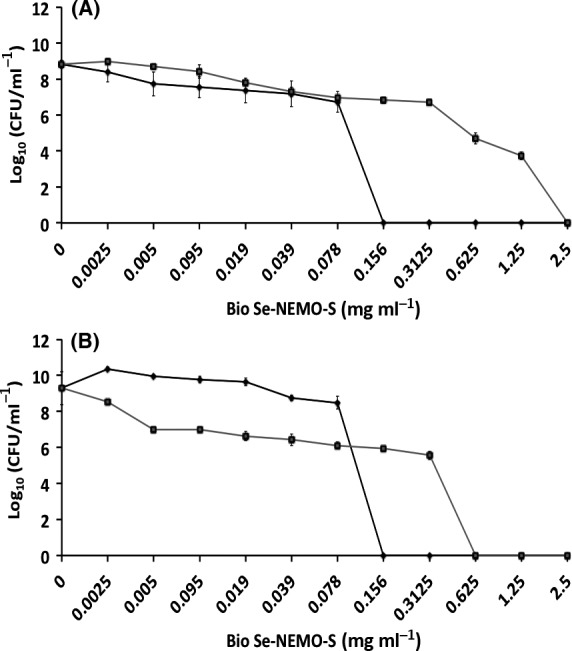
Minimal Biofilm Prevention Concentration (MBPC) assays of *Pseudomonas aeruginosa* NCTC 12934 (A) and *Staphylococcus aureus* ACTT 25923 (B) growing biofilms exposed for 24 h to 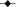 Bio Se‐NEMO‐S_6_ and 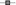 Bio Se‐NEMO‐S_24_. Error bars show the standard deviation.

**Figure 8:**
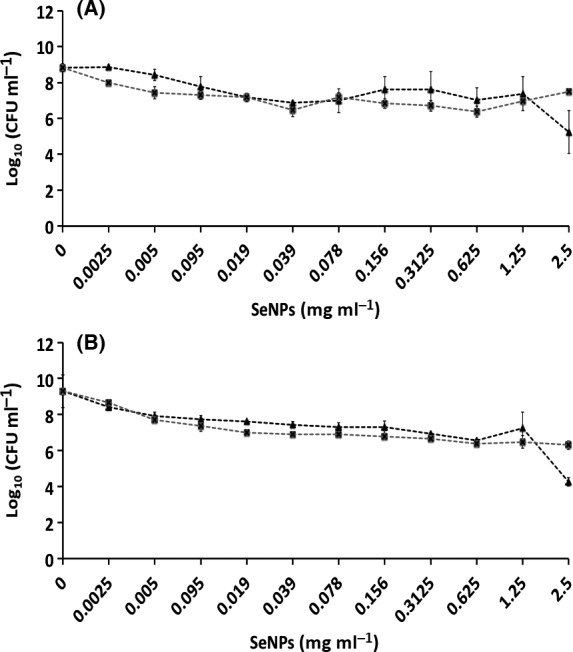
Minimal Biofilm Prevention Concentration (MBPC) assays of *Pseudomonas aeruginosa* NCTC 12934 (A) and *Staphylococcus aureus* ACTT 25923 (B) growing biofilms exposed for 24 h to 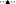 L‐cys SeNPs and 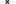 Asc SeNPs. Error bars show the standard deviation.

The CBDs were also used to evaluate the potential of either Bio Se‐NEMO‐S or SeNPs to inhibit biofilm formation by adding Se‐nanomaterials at the time of inoculation. In this assay, the bacterial cells must survive the stress/challenge of the biocide (Bio Se‐NEMO‐S or SeNPs) in the planktonic state long enough to initiate attachment and then proliferate as a biofilm. The data are plotted as number of *P. aeruginosa* (Figs 7A and 8A) or *S. aureus* (Figs 7B and 8B) viable cells (24 h) at varying concentrations of biocide.

For either Bio Se‐NEMO‐S or chemical SeNPs that demonstrated high antimicrobial activity, a Minimal Biofilm Prevention Concentration (MBPC), defined as the concentration after which an antimicrobial agent is able to totally inhibit and prevent cell attachment and biofilm proliferation, was established. Using this approach, an antibiofilm efficacy with differences in trends between chemical SeNPs and Bio Se‐NEMO‐S has been observed. Both Bio Se‐NEMO‐S_6_ and Bio Se‐NEMO‐S_24_ showed very good antimicrobial activity with defined MBPC that totally inhibited the establishment and growth of *P. aeruginosa* and *S. aureus* biofilms. In particular, Bio Se‐NEMO‐S_6_ has strong antibiofilm efficacy as well as Bio Se‐NEMO‐S_24_ against both tested strains, with MBPC values of 0.078 and 0.3125 mg ml^−1^ respectively (Fig. 7A and B). These results also showed that both L‐cys and Asc SeNPs are not able to completely prevent the formation of the pathogen indicator strain biofilms (Fig. 8A and B). However, L‐cys SeNPs still maintained antibiofilm activity, as they caused a decrease in the number of *P. aeruginosa* (3 log) and *S. aureus* (4 log) viable cells. Finally, Asc SeNPs showed a slight antimicrobial ability for the two tested strains. In particular, we observed a 1 log reduction in the number of biofilm‐resident bacteria against *P. aeruginosa* and 2 log in the case of *S. aureus*. As chemically synthesized SeNPs show a moderate antimicrobial ability, MBPC values were determined only for Bio Se‐NEMO‐S.

#### Efficacy on planktonic cultures

**Figure 9:**
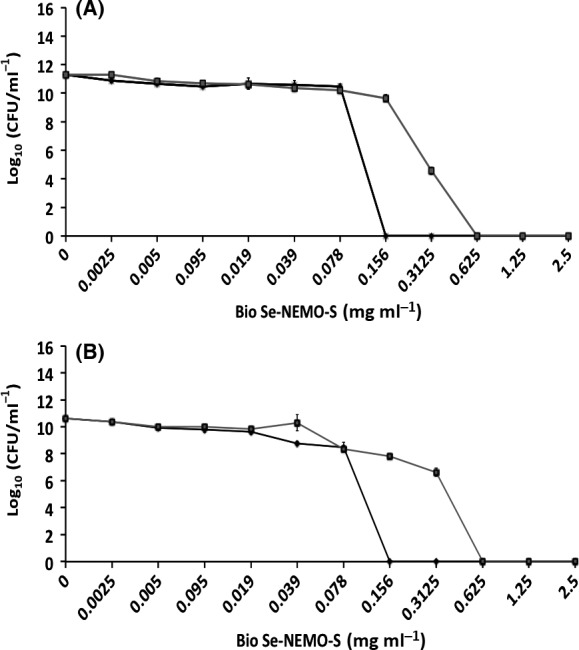
Minimal Inhibition Concentration (MIC) assays of *Pseudomonas aeruginosa* NCTC 12934 (A) and *Staphylococcus aureus* ACTT 25923 (B) growing planktonic cells exposed for 24 h to 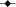 Bio Se‐NEMO‐S_6_ and 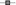 Bio Se‐NEMO‐S_24_. Error bars show the standard deviation.

**Figure 10:**
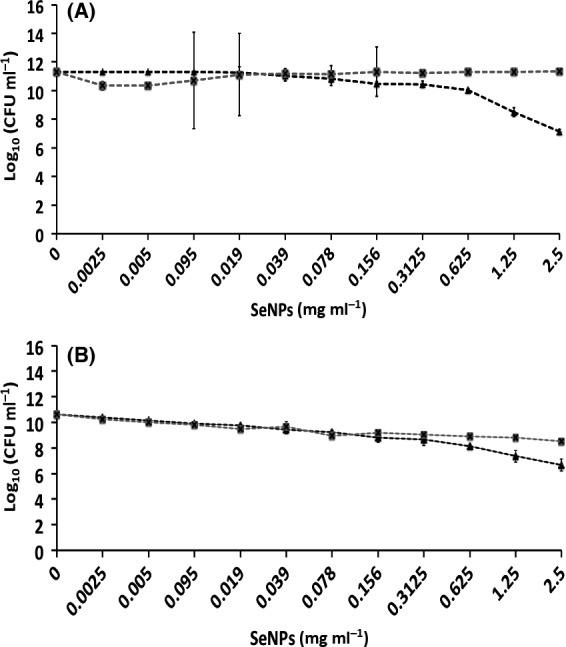
Minimal Inhibition Concentration (MIC) assays of *Pseudomonas aeruginosa* NCTC 12934 (A) and *Staphylococcus aureus* ACTT 25923 (B) growing planktonic cells exposed for 24 h to 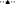 L‐cys SeNPs and 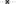 Asc SeNPs. Error bars show the standard deviation.

Efficacy of the both Bio Se‐NEMO‐S and chemical SeNPs against planktonic cells of *P. aeruginosa* (Figs 9A and 10A) and *S. aureus* (Figs 9B and 10B) was also tested. Results demonstrated a strong antimicrobial effect of Bio Se‐NEMO‐S on planktonic cultures of the tested strains. In this case, a Minimal Inhibitory Concentration (MIC), that is the concentration of an antimicrobial agent after which there is no planktonic cells growth, has been established for Bio Se‐NEMO‐S. Particularly, the MIC values of Bio Se‐NEMO‐S were equal to the MBPC values established for inhibiting biofilm growth, being 0.078 mg ml^−1^ for Bio Se‐NEMO‐S_6_ and 0.3125 mg ml^−1^ for Bio Se‐NEMO‐S_24_ against both *P. aeruginosa* and *S. aureus* cultures (Fig. 9A and B). Among the chemically synthesized SeNPs, L‐cys SeNPs reveal a moderate antimicrobial activity at the highest tested concentrations (1.25–2.5 mg ml^−1^) with a 4 log decrease in the number of *P. aeruginosa* and *S. aureus* growing cells (Fig. 10A and B). Moreover, Asc SeNPs display a slight efficacy only against *S. aureus* planktonic population (2 log reduction), without any decrease in the number of *P. aeruginosa* cells (Fig. 10A and B). Due to their moderate antimicrobial efficacy, no MIC values have been established for chemical SeNPs.

### Confocal Laser Scanning Microscopy (CLSM) analysis

**Figure 11:**
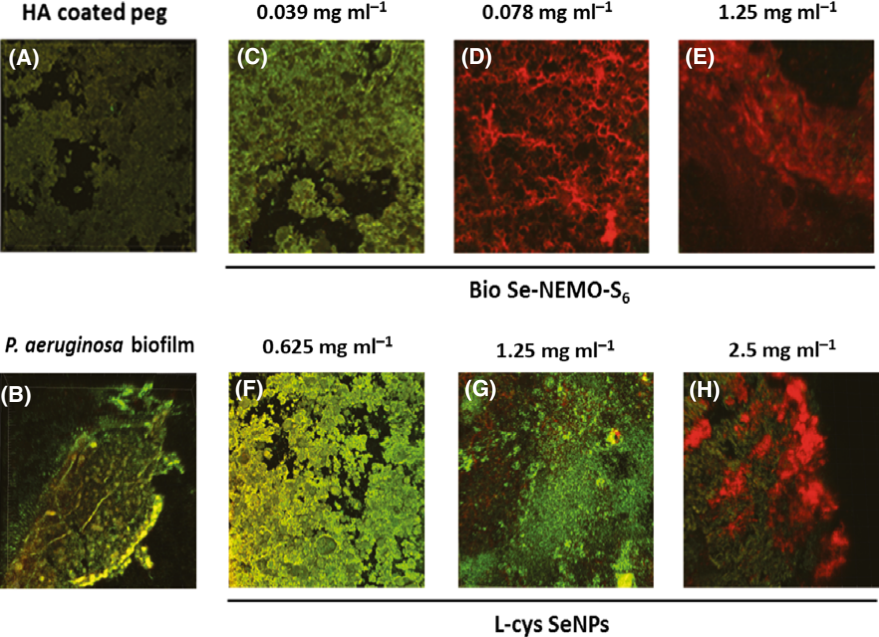
Confocal Laser Scanning Microscopy (CLSM) of HA‐coated peg (A), *Pseudomonas aeruginosa* biofilm grown onto HA‐coated peg (B) and in the presence of bio6 SeNPs at the concentration of 0.039 mg ml^−1^ (C), 0.078 mg ml^−1^ (MBBC) (D), 1.25 mg ml^−1^ (E), or in the presence of L‐cys SeNPs at the concentration of 0.625 mg ml^−1^ (F), 1.25 mg ml^−1^ (G), 2.5 mg ml^−1^ (H).

**Figure 12:**
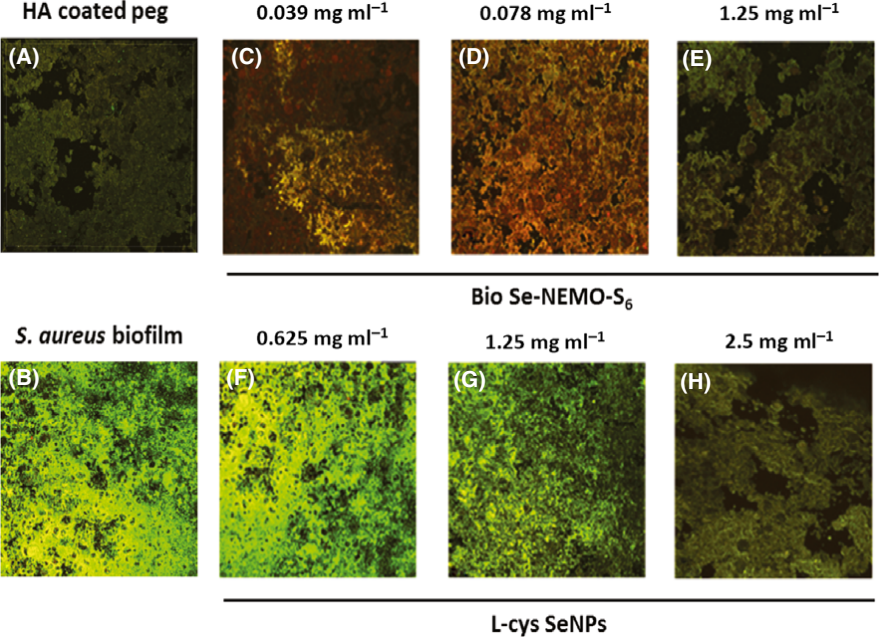
Confocal Laser Scanning Microscopy (CLSM) of HA‐coated peg (A), *Staphylococcus aureus* biofilm grown onto HA‐coated peg (B) and in the presence of bio6 SeNPs at the concentration of 0.039 mg ml^−1^ (C), 0.078 mg ml^−1^ (MBBC) (D), 1.25 mg ml^−1^ (E), or in the presence of L‐cys SeNPs at the concentration of 0.625 mg ml^−1^ (F), 1.25 mg ml^−1^ (G), 2.5 mg ml^−1^ (H).

*P. aeruginosa* (Fig. 11) and *S. aureus* (Fig. 12) biofilms growing on HA‐coated pegs in the presence of either Bio Se‐NEMO‐S or chemical SeNPs have been observed by performing CLSM analyses. We chose to analyse Bio Se‐NEMO‐S_6_, as this nanomaterial possessed the strongest antibiofilm activity, making a comparison with L‐cys SeNPs, which showed the highest antimicrobial efficacy among the tested chemical SeNPs. Specifically, we analysed three samples of *P. aeruginosa* and *S. aureus* growing biofilms exposed to the different Bio Se‐NEMO‐S_6_ SeNP concentrations of 0.039, 0.078 mg ml^−1^ (established MBPC) and 1.25 mg ml^−1^. Due to the absence of an defined MBPC for L‐cys SeNPs, the antimicrobial effect of these NPs has been evaluated exposing *P. aeruginosa* and *S. aureus* growing biofilms to the three highest SeNP concentrations of the tested range: 0.625, 1.25 and 2.5 mg ml^−1^. Samples were stained using Live/Dead^®^ BacLight™ staining kit, as described in the Methods section. Due to the ion exchange chemistry of HA, the cations of the stain were able to bind to HA, resulting in a dark green or red background noise (Figs 11A and 12A), which was different from the brilliant and strong fluorescence corresponding to the stained cells.

*P. aeruginosa* biofilms grown in the presence of the lowest concentrations of Bio Se‐NEMO‐S_6_ (0.039 mg ml^−1^) or L‐cys SeNPs (0.625 mg ml^−1^; Fig. 11C and F) were characterized by a strong green‐fluorescent signal, indicating a mature and vibrant biofilm. However, *P. aeruginosa* biofilm exposed to the action of Bio Se‐NEMO‐S_6_ showed a reduced consistency (375 μm), while L‐cys SeNPs had a slight effect on biofilm thickness (415 μm), being comparable to the non‐treated one (450 μm; Fig. 11B). Observations are drastically different considering *P. aeruginosa* biofilms grown in the presence of 0.078 mg ml^−1^ (MPBC) or 1.25 mg ml^−1^ of Bio Se‐NEMO‐S_6_ (Fig. 11D and G), which showed strong and brilliant red colour, indicating not viable cells. Moreover, the thickness of these biofilms dropped to values of 150.4 μm for 0.078 mg ml^−1^ or 75.3 μm in the case of the highest tested concentration (1.25 mg ml^−1^). By contrast, samples grown with 1.25 mg ml^−1^ or 2.5 mg ml^−1^ L‐cys SeNPs had both green‐ and red‐fluorescent signals (Fig. 11E and H), demonstrating that there was a coexistence of live and dead bacterial cells. Furthermore, analysis of these samples showed the presence of thick biofilms for both tested concentrations of chemical SeNPs, being 223 μm (1.25 mg ml^−1^) and 185,5 μm (2.5 mg ml^−1^).

*S. aureus* biofilm grown on HA‐coated peg (Fig. 12B) appeared to be thinner (285 μm) as compared with that of *P. aeruginosa* (450 μm). A slight fluorescent signal was observed when *S. aureus* cells growing as a biofilm were exposed to 0.039 or 0.078 mg ml^−1^ (MPBC) of Bio Se‐NEMO‐S_6_ (Fig. 12C and D), resulting in an evaluated thickness of 94.2 and 2.5 μm respectively. This value decreased till 0.95 μm when *S. aureus* biofilm was grown in the presence of 1.25 mg ml^−1^ of Bio Se‐NEMO‐S_6_, with a fluorescence emission comparable to the HA‐coated peg itself (Fig. 12A and E). On the opposite, the lowest concentration of L‐cys SeNPs (0.625 mg ml^−1^) slightly affected *S. aureus* biofilm growth, as highlighted by the strong and green fluorescence due to the presence of a live and thicker biofilm (192.7 μm; Fig. 12F). Moreover, when *S. aureus* cells were incubated with 1.25 or 2.5 mg ml^−1^ of L‐cys SeNPs, the growing biofilm displayed a thickness of 114 or 25 μm respectively.

## Discussion

In this study, we analyse the potential of either biogenic SeNPs produced by *B. mycoides* SelTE01 (Lampis *et al*., 2014) indicated as Bio Se‐NEMO‐S or chemically synthesized SeNPs (L‐cys SeNPs and Asc SeNPs) as antimicrobial agents against pathogenic indicator strains *P. aeruginosa* NCTC 12934 and *S. aureus* ATCC 25923 grown on CBDs coated with HA, which is an important bioactive ceramic material used in orthopaedic and dental surgery (Gong *et al*., 2015). Considering the possible medical application of such nanomaterials, the toxicity of either Bio Se‐NEMO‐S produced by *B. mycoides* SelTE01 or chemical SeNPs towards human cells has been investigated by Cremonini *et al*. (2016), showing no cytotoxicity and biocompatibility with human cell lines (i.e. dendritic cells and fibroblasts).

In the evaluation of Se‐nanomaterials’ ability to eradicate both established *P. aeruginosa* and *S. aureus* biofilms, either Bio Se‐NEMO‐S or chemical SeNPs led to a decrease in the number of viable cells. Bio Se‐NEMO‐S_24_ SeNPs showed the strongest antimicrobial activity at concentration ranging from 0.625 to 2.5 mg ml^−1^ against *S. aureus* grown biofilm. In this respect, the moderate efficacy of antimicrobials towards an already established biofilm is due to the complexity of the biofilm structure itself. In particular, bacterial cells within a biofilm are surrounded by an extracellular polymeric substance (EPS), which provides structural stability, as well as acts like a barrier against antibiotics, metallic ions and bactericides (Harrison *et al*., 2004). Moreover, a subpopulation of biofilm‐resident bacteria behaves as persister cells, which are dormant and metabolically inactive (Hobby *et al*., 1942). These cells were observed for the first time by Hobby *et al*. (1942) and Bigger (1944) studying the effect of penicillin against *S. aureus* and *S. pyogenes* strains respectively. In both cases, some cells were able to survive in the presence of the antibiotic without undergoing genetic change. In this regard, persister cells should be considered as a subpopulation of bacteria with tolerance to antibiotics (Bigger, 1944). The presence of both EPS and persister cells inside a biofilm structure can explain the modest antimicrobial activity of tested Se‐nanomaterials in the eradication of *P. aeruginosa* and *S. aureus* established biofilms.

In the present study, the antimicrobial efficacy of Bio Se‐NEMO‐S and chemically synthesized SeNPs has been also evaluated towards *P. aeruginosa* and *S. aureus* cells growing under either planktonic or biofilm conditions. In both scenarios, Bio Se‐NEMO‐S highlighted a great and equivalent antimicrobial ability against the tested strains. Particularly, growth of planktonic cells and biofilm formation are inhibited at the same concentrations of Bio Se‐NEMO‐S_6_ (0.078 mg ml^−1^) and Bio Se‐NEMO_24_ (0.3125 mg ml^−1^) for both *P. aeruginosa* and *S. aureus* strains respectively. Notably, Bio Se‐NEMO‐S_6_ were more efficient than Bio Se‐NEMO‐S_24_ in preventing the biofilm establishment and growth. Zonaro *et al*. (2015) have observed a similar behaviour for SeNPs produced by *Stenotrophomonas maltophilia* SelTE02, when it was exposed to Na_2_SeO_3_ for 6 or 24 h. In this case, those produced after 6 h showed a stronger antimicrobial activity against *P. aeruginosa* PAO1, *S. aureus* ATCC 25923 and *E. coli* JM109 strains rather than those obtained after 24 h (Zonaro *et al*., 2015). This diversity in action for biogenic SeNPs is ascribed to the different size of SeNPs themselves (Zonaro *et al*., 2015). Similar results have been observed by Lu *et al*. (2013) in the evaluation of AgNPs’ action as antimicrobial agents against oral anaerobic pathogenic bacteria. Lu *et al*. (2013) indicated a size‐dependent mechanism of AgNPs’ action, by which smaller NPs have higher antimicrobial effect. In our study, the same observation is supported by DLS analyses, in which Bio Se‐NEMO‐S_6_ showed a smaller hydrodynamic diameter (160 nm) as compared to Bio Se‐NEMO‐S_24_ (209.1 nm). Furthermore, the Bio Se‐NEMO‐S sizes suggested the existence of a time‐dependent mechanism of biogenic SeNPs’ production. In this regard, improving the exposure time of *B. mycoides* SelTE01 to the selenium precursor (Na_2_SeO_3_) resulted in the production of bigger Se‐nanomaterials, as already described by Lampis *et al*. (2014). As Bio Se‐NEMO‐S_6_ showed the highest antimicrobial ability among all tested NPs, we further performed CLSM analyses to evaluate the fitness of *P. aeruginosa* and *S. aureus* cells growing as a biofilm in the presence of this class of biogenic SeNPs. As we observed a reduction in the thickness of both studied biofilms, CLSM observations correlate with our kill curve results. Nevertheless, *P. aeruginosa* and *S. aureus* growing biofilms showed completely different fluorescence emission at the efficient concentration of Bio Se‐NEMO‐S_6_, resulting in a strong red‐fluorescent signal or in one comparable to the HA‐coated peg control respectively.

These data suggest two possible distinct modes of antimicrobial action of Bio Se‐NEMO‐S_6_ depending on the pathogenic indicator strain analysed. The presence of large amount of red cells in *P. aeruginosa* biofilm underlines Bio Se‐NEMO_6_ ability to kill bacterial cells or to limit the number of viable cells. At the same time, the absence of thick *S. aureus* biofilm highlights Bio Se‐NEMO‐S_6_ action in inhibiting irreversible attachment of bacterial cells to form a biofilm.

Chemically synthesized SeNPs showed less antimicrobial activity towards both *P. aeruginosa* and *S. aureus* planktonic cell growth and biofilm formation compared to those biogenically produced. Tran and Webster (2011) observed similar results in the evaluation of the antimicrobial efficacy of chemical SeNPs against planktonic cells of *S. aureus*. CLSM observations of both *P. aeruginosa* and *S. aureus* growing biofilms in the presence of L‐cys SeNPs confirm our kill curve results. Even at the highest L‐cys SeNPs tested concentration, *P. aeruginosa* cells exhibited a green‐fluorescence emission, indicating the presence of live cells in the biofilm. Moreover, an established and thick biofilm was observed in the case of *S. aureus* cells incubated in the presence of 1.25 mg ml^−1^ L‐cys SeNPs, while a slight inhibition is revealed at the highest tested concentration (2.5 mg ml^−1^).

The higher antimicrobial ability of Bio Se‐NEMO‐s as compared to those chemically synthesized could be explained by the presence of an embedding organic matrix that surrounded these nanomaterials, as confirmed by TEM analyses, EDX spectra and zeta potential measurements. Specifically, L‐cys and Asc SeNPs TEM images underlined evidence of SeNPs in a bright and white field, while both Bio Se‐NEMO‐S were characterized by a grey slightly electron‐dense surrounding material. EDX analyses of all Se‐nanomaterials highlighted the presence of carbon, oxygen, phosphorous and sulphur, along with selenium. Considering the synthesis procedure of chemical SeNPs, the detection of these elements in EDX analyses is explained by the use of either L‐cysteine, rich in C, O and S, or ascorbic acid, rich in C and O, for the production (Zhang *et al*., 2004; Li *et al*., 2010). In the case of Bio Se‐NEMO‐S, the existence of C, O, P and S suggested the evidence of selenium nanoparticles surrounded by an organic material. These observations were validated by zeta potential measurements, in which both Bio Se‐NEMO‐S and chemical SeNPs showed strongly negative surface charge values. Considering that elemental selenium (Se^0^) does not have a net charge, these results suggested that the stability of the Se‐nanomaterials in solution is facilitated by the presence of negatively charged organic chemical groups. The association of SeNPs produced by *B. mycoides* SelTE01 with a surrounding material has been observed also by Lampis *et al*. (2014), which ascribed the nature of this external layer to the adhesion of proteins to SeNPs. The existence of proteins associated with biogenic SeNPs has been established and studied for other bacterial strains able to reduce selenium oxyanions to elemental selenium (Se^0^). Debieux *et al*. (2011) have identified some proteins associated with SeNPs produced by *Thauera selenatis* that played an important role in stabilizing the formation of NPs, while Lenz *et al*. (2011) highlighted that SeNPs produced by *Bacillus selenatarsenatis* and *Sulfurospirillum barnesii* were characterized by the association with several high‐affinity proteins. Recently, Zonaro *et al*. (2015) have observed the presence of several organic compounds surrounding biogenic SeNPs produced by *S. maltophilia* SelTE02, which have been characterized as carbohydrates, lipids and proteins by Lampis *et al*. (2016) using FT‐IR analyses. This evidence complements our characterization analyses regarding the presence of associated organic material surrounding biogenic SeNPs.

In our study, despite DLS results that showed a defined size for all the studied Se‐nanomaterials, TEM analyses highlighted the presence of smaller yet monodispersed SeNPs in Bio Se‐NEMO‐S samples of less than 100 nm in size. Probably, this discrepancy in size between DLS measurements and TEM observations is due to the contribution of the organic surrounding material in which biogenic SeNPs are embedded.

## Conclusions

In our study, we evaluated the antimicrobial efficacy of biogenic SeNPs produced by *B. mycoides* SelTE01 (Bio Se‐NEMO‐S) compared to chemical SeNPs against two pathogenic indicator strains, *P. aeruginosa* NCTC 12934 and *S. aureus* ATCC 25923, grown onto HA‐coated pegs. Bio Se‐NEMO‐S showed higher antimicrobial efficacy as compared to chemically synthesized SeNPs in all three evaluated scenarios for both tested strains. Difference in antimicrobial ability of these Se‐nanomaterials suggests either distinct chemistry or the involvement of the organic surrounding material associated with the SeNPs (Bio Se‐NEMO‐S) originating from *Bacillus mycoides* SelTE01. Finally, this study demonstrates good promise of utilizing Bio Se‐NEMO‐S as antimicrobial agents to inhibit biofilm formation onto HA‐coated pegs.

## Experimental procedures

### Materials, media and organisms used

*Bacillus mycoides* SelTE01 has been isolated previously from the rhizosphere of the Se‐hyperaccumulating plant *Astragalus bisulcatus* (Lampis *et al*., 2014). The two defined indicator strains used are *P. aeruginosa* NCTC 12934 and *S. aureus* ATCC 25923. Nutrient broth, agar and sodium chloride (NaCl) were obtained from Oxoid, Basingstoke Hampshire, England. Sodium selenite (Na_2_SeO_3_), octanol, L‐cysteine, ascorbic acid, L‐histidine, reduced glutathione were obtained from Sigma Aldrich, St. Louis, MO, USA. Tris–HCl 1.5 mM was made by adding 0.091 g of Trizma base (Sigma Aldrich) to 500 ml of water, and then, using HCl from Sigma Aldrich, pH was adjusted to 7.0. HA‐coated Calgary Biofilm Device (CBD) plates or MBEC™was obtained from Innovotech, Edmonton, AB, Canada.

### Methods

#### Chemical synthesis of SeNPs

SeNPs were chemically synthesized by adding 500 μl of 100 mM Na_2_SeO_3_ to 500 μl of 50 mM L‐cysteine as the reducing agent and 1 ml of milli‐Q water (L‐cys SeNPs) (Li *et al*., 2010), or using 1 ml of 30 mM ascorbic acid (reducing agent) to 200 μl of 100 mM Na_2_SeO_3_ and 800 μl of milli‐Q water (Asc SeNPs; Zhang *et al*., 2004).

#### Biogenic SeNPs (Bio Se‐NEMO) synthesis

Following the protocol established by Lampis *et al*. (2014), *B. mycoides* SelTE01 was grown in culture tubes with 4 ml of nutrient broth medium for a minimum 24 h at 130 rpm. These cultures were used to inoculate 1‐l flasks containing 400 ml of nutrient broth medium and 8 ml of filter sterilized Na_2_SeO_3_ (100 mM). The cultures were subsequently incubated at 27°C at 130 rpm, for 24 h. During this incubation, reduction of SeO_3_^2−^ ions to Se^0^ was observed visually through the formation of a red colour solution, typical of colloidal elemental selenium. Samples were collected after 6 and 24 h.

To extract the Se‐nanomaterial from *B. mycoides* cultures, the contents of each flask were divided into eight 50‐ml conical tubes and collected by centrifugation at 10 000 × *g* for 10 min. Pellets were washed twice with 0.9% NaCl solution, resuspended in Tris/HCl buffer (pH 8.2) and then disrupted by ultrasonication at 100 W for 5 min. The suspension was then centrifuged at 10 000 × *g* for 30 min to separate disrupted cells (pellet) from nanomaterials (supernatant), which were recovered after centrifugation at 40 000 × *g* for 30 min, washed twice with saline solution (0.9% NaCl) and resuspended in deionized water. This washed suspended material is what we characterize here and is defined as Spherical Bio Se‐Nanostructures Embedded in an Organic material (Bio Se‐NEMO‐S).

#### Characterization of Bio Se‐NEMO‐S and SeNPs

Size, polydispersity index (PDI) and surface charge of chemical and biogenic SeNPs (Bio Se‐NEMO‐S) have been studied by evaluating their dynamic light scattering (DLS) and measuring their zeta potential. DLS measurements for all Se‐nanomaterials have been evaluated using Zen 3600 Zetasizer Nano ZS from Malvern Instruments (Worcestershire, UK) (Cremonini *et al*., 2016). Hydrodynamic diameter and PDI values were obtained using the software provided by the Malvern with the instrument. All the samples were then transferred to a quartz cuvette (10 mm path length), and the zeta potential at pH 7 has been measured at 25°C using the Malvern software. TEM analyses have been carried out to study morphology, size and shape for both the chemical and biogenic (Bio Se‐NEMO‐S) Se‐nanomaterials. For TEM observations, 5 μl of each Se‐nanomaterials was mounted on carbon‐coated copper grids (CF300‐CU; Electron Microscopy Sciences, Hatfield, PA, USA). Then, samples were air‐dried and visualized using a Hitachi H7650 TEM. Finally, EDX spectra have been performed using an XL30 ESEM (FEI) equipped with an EDX micro‐analytical system. Each sample was fixed, dehydrated through an increasing ethanol concentration series and dried in liquid CO_2_. The Se‐nanomaterials were then mounted on metallic stubs and directly observed to perform EDX analyses.

#### Measuring the biocidal activity of Bio Se‐NEMO‐S and chemical SeNPs

Kill curve assays were performed to evaluate the antimicrobial activity of either Bio Se‐NEMO‐S or chemical SeNPs. Here, we used the Calgary Biofilm Device (CBD) that is a 96‐well microtitre plate lid with pegs protruding into the microtitre plate wells (Ceri *et al*., 2001). The use of this device to culture the bacteria permits high‐throughput challenging with chemically synthesized SeNPs and Bio Se‐NEMO‐S. In this study, the CBD pegs had been pre‐treated with a HA coating, generating a system relevant to dental and orthopaedic medicine. The assay was conducted using *P. aeruginosa* (NCTC 12934) and *S. aureus* (ATCC 25923), both established laboratory indicator strains of pathogens.

The CBD was inoculated with *P. aeruginosa* or *S. aureus* cells grown in nutrient broth medium along with solutions of either Bio Se‐NEMO‐S or chemical SeNPs at various concentrations. We tested two biogenic Se‐nanomaterials produced by *B. mycoides* SelTE01 after 6 h (Bio Se‐NEMO‐S_6_) and 24 h (Bio Se‐NEMO‐S_24_) of Na_2_SeO_3_ exposure. Additionally, we tested two chemically generated SeNPs made using L‐cysteine (L‐cys SeNPs; Li *et al*., 2010) or ascorbic acid (Asc SeNPs; Zhang *et al*., 2004) as reducing agents. Inoculum was prepared by resuspending biomass collected from a second subculture on a nutrient broth–agar plate in sterile distilled deionized water. Turbidity of the solution was adjusted to achieve an equivalent optical density of 0.5 McFarland standardized suspension. This suspension was then diluted in nutrient broth medium to achieve an approximate cell density of 10^5^ per ml. Overall, we examined three different scenarios:

- The ability of Bio Se‐NEMO‐S and SeNPs to eradicate 24‐h pre‐grown biofilms (minimal biofilm eradication concentration; MBEC).
- The ability of Bio Se‐NEMO‐S and SeNPs to prevent the formation of a biofilm (minimal biofilm prevention concentration; MBPC).
- The ability of Bio Se‐NEMO‐S and SeNPs to inhibit planktonic cells growth (minimal inhibitory concentration; MIC)

For i, the CBD was inoculated with *P. aeruginosa* or *S. aureus* strains at a 0.5 McFarland standard concentration in nutrient broth medium and placed in a gyrorotary shaker operating at 150 rpm, 37°C for 24 h. Following the establishment of a biofilm, solutions containing various concentrations of each tested Se‐nanomaterials were added to the wells in twofold serial dilutions between 2.5 and 0.0025 mg ml^−1^. Experiments were performed in triplicate. The inoculated CBDs were placed on a shaker at 150 rpm, 37°C for 24 h.

For ii, CBDs were inoculated with a 0.5 McFarland standard of the bacterial strains into nutrient broth medium and Bio Se‐NEMO‐S or chemical SeNPs solutions were added as described in scenario i, allowing biofilm growth for 24 h.

To collect biofilm biomass from scenarios i and ii, peg lids were rinsed twice with 0.9% NaCl to remove loosely bound cells and then placed into 96‐well recovery plates containing 180 μl each of a mixture 1:100 of universal neutralizer solution and nutrient broth medium. Finally, we sonicated for 30 minutes to remove biofilm biomass from the peg surface.

For iii, 96‐well plates containing nutrient medium and Se‐nanomaterial solutions described in scenario i were inoculated with *P. aeruginosa* and *S. aureus* cells. The inoculated plates were incubated at 37°C with shaking (150 rpm) for 24 h.

Biofilm and planktonic cells exposed to either Bio Se‐NEMO‐S or chemical SeNPs from all three scenarios were collected and serially diluted into 0.9% saline solution. Viable cell numbers were determined using spot plates count. All assays were conducted using three biological replicates. Data are reported as means with standard deviations.

#### Confocal laser scanning microscopy (CLSM)

A LEICA model DM IRE2 microscope was used to collect confocal images further processed using imaris ×64 (Bitplane, Concord, MA, USA) software. Briefly, hydroxyapatite‐coated CBD pegs containing biofilms were aseptically removed from the lid using alcohol‐flamed pliers. The collected pegs were rinsed twice with 200 μl of 0.9% NaCl solution in a microtiter plate and stained using the Live/Dead^®^ BacLight™ stain (Molecular Probes, Ontario, Canada) for 30 minutes prior to visualization (Harrison *et al*., 2006; Cerca *et al*., 2012). This kit contains SYTO 9 green‐fluorescent nucleic acid stain and propidium iodide, which is a red‐fluorescent dye (Dailey, 2006). These stains have diverse spectral characteristics, and they are able to differentially penetrate bacterial cells. SYTO 9 generally labels all bacterial cells in population, while propidium iodide penetrates only bacterial cells with damaged membranes (Dailey, 2006). The thickness of the biofilms has been estimated through obtaining high‐resolution optical images with depth selectivity, allowing 3D images of complex biological samples (Dailey, 2006).

## Conflict of interest

The authors have no conflict of interest to declare.
